# The Application of Platelet-Rich Plasma for Patients Following Total Joint Replacement: A Meta-Analysis of Randomized Controlled Trials and Systematic Review

**DOI:** 10.3389/fsurg.2022.922637

**Published:** 2022-07-04

**Authors:** Hongxin Shu, Zhenjun Huang, Xinyan Bai, Zhiyu Xia, Nanye Wang, Xiaoling Fu, Xigao Cheng, Bin Zhou

**Affiliations:** Department of Orthopedics, The Second Affiliated Hospital of Nanchang University, Nanchang, China

**Keywords:** meta-analysis, randomized controlled trial, total joint replacement, total knee arthroplasty, total hip arthroplasty, platelet-rich plasma

## Abstract

**Background:**

The clinical efficacy of platelet-rich plasma (PRP) in the treatment of total joint replacement (TJR) remains inconclusive. In this paper, systematic review and meta-analysis was adopted to assess the efficacy of using PRP for the treatment of TJR.

**Methods:**

A comprehensive search of Medline, Embase, and Cochrane library databases for randomized controlled trial (RCT) articles recording data of PRP for TJR was conducted from inception to February 2022. Outcomes concerned were pain, range of motion (ROM), WOMAC score, length of hospital stay (LOS), hemoglobin (Hb) drop, total blood loss, wound healing rate, and wound infection. The methodological quality of the included RCTs was evaluated by using the Cochrane Risk of Bias Tool 2.0 (RoB 2.0). The Grading of Recommendations Assessment, Development, and Evaluation (GRADE) was utilized to assess the level of evidence for the outcomes. Subgroup analysis was conducted according to the type of TJR.

**Results:**

Ten RCTs were included in the meta-analysis. In the TKA subgroup, the available data demonstrated that there were significant differences in the outcomes of pain and Hb drop, while it was the opposite of ROM, WOMAC score, LOS, total blood loss, wound healing rate, and wound infection. In the THA subgroup, no significant differences could be seen between two groups in the outcomes of LOS and wound infection. However, the PRP group gained a higher wound healing rate in the THA subgroup.

**Conclusion:**

The application of PRP did not reduce blood loss but improved the wound healing rate. However, more prospective and multicenter studies are warranted to confirm these results.

## Background

Total joint replacement (TJR) is a common surgical procedure in which the weight-bearing surface of a joint is replaced to restore its capacity and function (1–5). During the operation, TJR can cause many complications, including blood loss, deep vein thrombosis, and wound complications (5). These disorders have a poor prognosis and can cause severe pain, which increases the length of hospital stay (LOS), medical costs, and even the risk of deep vein thrombosis (DVT) (6). Nowadays, multiple approaches have been used to manage complications arising after TJR, including fibrin tissue adhesive, epidural infusion, and oral opioids (3, 7, 8). However, the treatment outcome is not satisfactory and is accompanied by side effects (9).

Platelet-rich plasma (PRP) has been increasingly used in the field of sports injuries and has attracted extensive attention due to its high safety, simple preparation, and ease of extraction (10). PRP is a highly concentrated platelet solution extracted from autologous whole blood by centrifugation. PRP can release high concentrations of autogenous growth factors, including transforming growth factor *β*1 (TGF-*β*1), platelet-derived growth factor, insulin-like growth factor (IGF), and epidermal growth factor (EGF), which can promote chondrocyte proliferation and vascular growth to accelerate wound site repair (11). A large amount of fibrin also contributes to wound repair, which facilitates wound contraction and provides scaffolding (12, 13). Previous meta-analysis demonstrated that PRP does accelerate wound healing for diabetic foot ulcers and venous ulcers (14). However, the clinical evidence for TJR was lacking.

In recent years, an increasing number of studies on the use of PRP in TJR have been conducted, but many clinical studies have not drawn definitive conclusions on the efficacy and safety of PRP. A previous meta-analysis (15) concluded that there was no significant difference in pain at 24 h and 48 h, which is contradictory to a recent study (16). Ma J et al (17) performed a meta-analysis including six randomized controlled trials (RCTs), demonstrating that PRP reduced blood loss after total knee arthroplasty (TKA). However, data from subsequent studies on this topic revealed that there was no statistical difference between the PRP group and the control group (18–20). RCTs published in recent years may alter previous conclusions on the effect of PRP. Hence, this systematic review and meta-analysis related to the use of topical PRP for TJR was performed to explore the potential clinical values of PRP.

## Material and Methods

This systematic review was conducted by following Preferred Reporting Items for Systematic Reviews and Meta-Analyses (PRISMA) (21). Because it was a review of the existing literature, and there was no registered protocol, ethical approval was not necessary.

### Search Strategy

Relevant randomized controlled trials (RCTs) were identified from databases Medline, the Cochrane library, and Embase. The search strategy for Medline was as follows: ((“Platelet-rich plasma” or “PRP”) and (“Arthroplasty, Replacement, Hip” or “THA” or “THA” or “Total Joint Arthroplasty” or “Arthroplasty, Replacement, Knee” or “TKA”)). The last search date was February 2, 2022. We manually searched for eligible references by reading the title and abstract. In addition, we manually screened previous reviews and reference lists of relevant studies to broaden the search.

#### Study Selection

After downloading all citations, RCTs were reviewed by two independent investigators (HS, ZH), and any disagreements were resolved by a third investigator (BZ). All RCTs meeting the following inclusion criteria were considered: patients (age>18 years) who underwent primary TJR; and PRP intervention in the experiment group. There were no restrictions on the year of publication, and language was limited to English. The following types were excluded: letter, case report, case series, review, non-RCT, and Quasi-RCT.

#### Data Extraction

The following items were extracted from eligible studies by two independent investigators (HS, ZH): name of first author, year of publication, country of origin, the number of patients in each group, the number of males in each group, mean (± standard deviation) age of patients per group, type of operation, type of prosthesis, type of PRP, preparation of PRP, and the dose of PRP. A standardized Microsoft Excel file was used to record the data and a third investigator (BZ) verified the collected data.

#### Critical Appraisal

Based on Cochrane Collaboration's tool, risk of bias 2.0 (RoB 2.0) (22) was assessed by two independent investigators (HS, ZH), and any disputes were resolved by another investigator (BZ). The overall level of evidence for each endpoint was evaluated by Grading of Recommendations dations Assessment, Development, and Evaluation (GRADE) (23).

#### Outcomes and Statistical Analysis

The outcomes of interest were pain, range of motion (ROM), Western Ontario and McMaster Osteoarthritis Index (WOMAC) score, length of hospital stay (LOS), hemoglobin (Hb) drop, total blood loss, wound healing rate, and wound infection. Visual analogue scale (VAS) was used to measure pain, which consisted of a scale of 1 to 10. All calculations were performed using STATA 17.0 software (StataCorp, College Station, TX). For continuous outcomes, mean difference (MD) was calculated, and the risk ratio (RR) was calculated for dichotomous outcomes. Heterogeneity was calculated using the I2 statistical and Chi-square test, indicating high heterogeneity when I2 was greater than 50%. In this case, a random-effects model was used; otherwise, we conducted the fixed-effects model. The p-value of less than 0.05 was defined as a significant difference. Summary effect measures were presented along with their corresponding 95% confidence intervals (CIs). Sensitivity analyses were conducted by omitting one study at a time, and subgroup analyses were performed to explore the source of bias according to the type of TJR. Egger’s test and Begg’s test were performed to assess publication bias (24, 25).

## Results

### Study Selection

**Figure 1:**
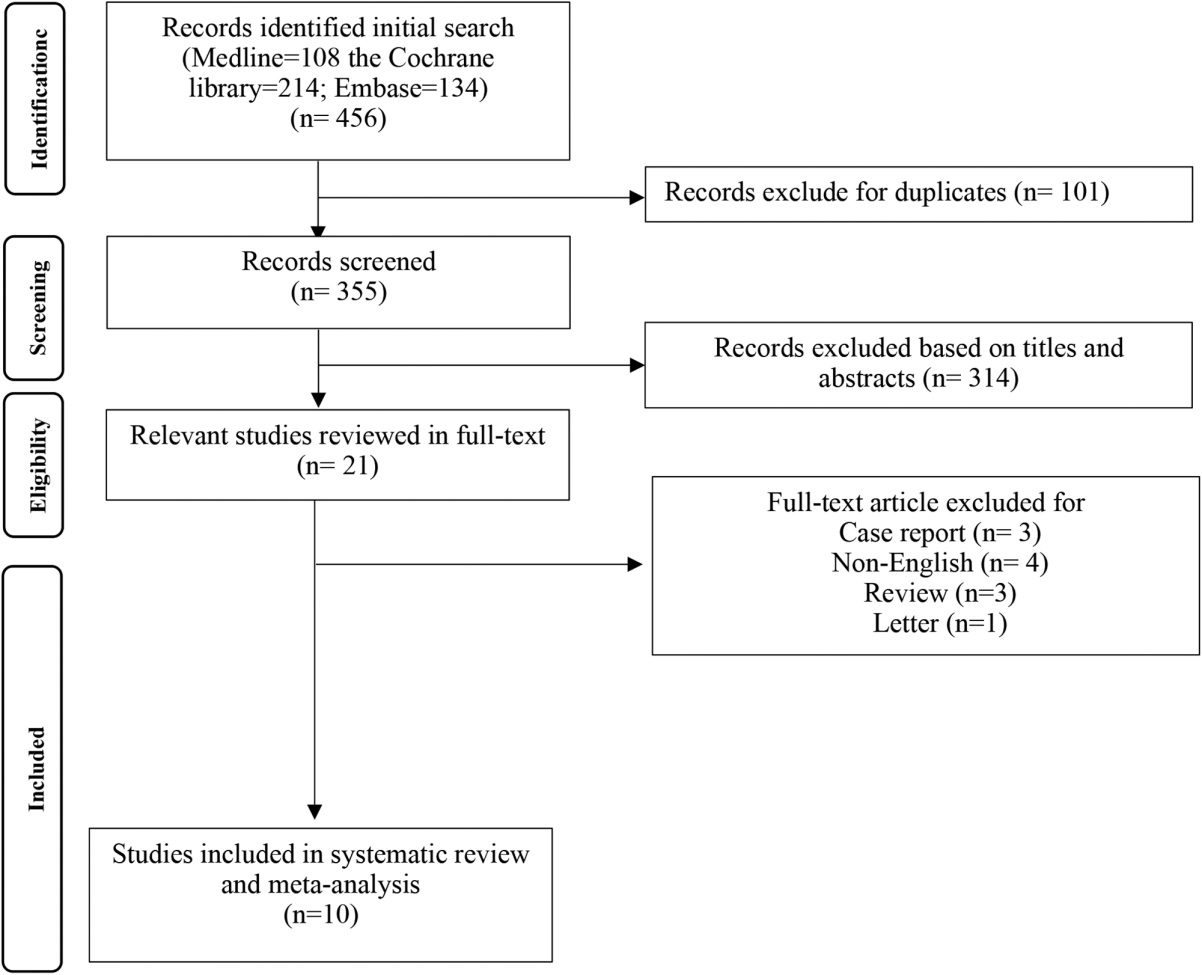
Process of study selection.

The literature search resulted in 456 hits, of which 101 were duplicates and were excluded. After screening based on titles and abstracts, the full text of 21 relevant studies was eligible. Three case reports, four non-English, three reviews, and one letter were excluded. Subsequently, a total of 10 origin RCTs (16, 18, 26–33) were included in the current systematic review (Figure 1).

### Study Characteristics

**Figure 2:**
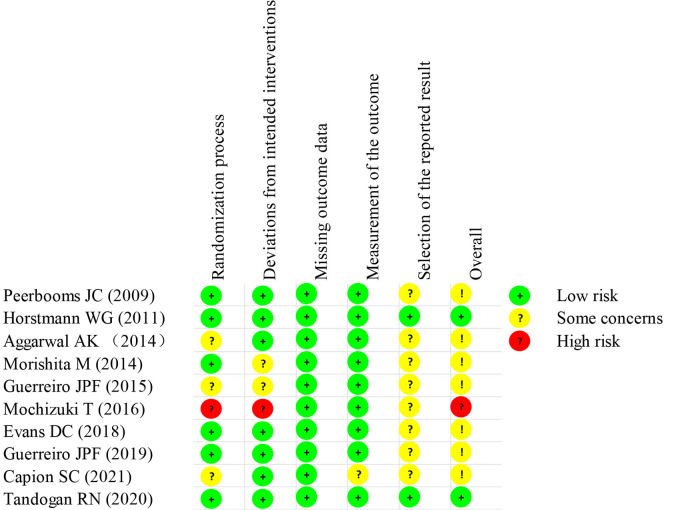
Risk of bias assessment of the included studies.

The studies are summarized in Table 1. A total of 772 patients who underwent TKA or total hip arthroplasty (THA) were included, of which 332 were in the PRP group. Eight studies reported the effect of PRP on TKA, one study reported that on THA, and one study reported that on TKA and THA. The studies were published between 2009 and 2021. The risk of bias is presented in Figure 2. One study (31) was defined as high bias and two studies (18, 27) were defined as low bias.

**Table 1:** Summary of included randomized controlled trials.

The first author	Country	Study design	No. patients (PRP/Control)	No. males (PRP/Control)	Age, mean (SD)		Follow-up, months	TKA/THA	Unilateral/bilateral	Type of prosthesis	Type of PRP; Product or Manual Protocol (No. of Spins)*	PRP dosage, mL
					PPR	Control						
Peerbooms JC (2009)	Netherlands	RCT	50/52	6/8	76 (4.1)	78 (5.2)	3	TKA	Yes/no	Cemented	LR-PRP; Biomet Biologics GPS	6
Horstmann WG (2011)	Netherlands	RCT	20/20	14/13	67 (6)	66 (6.75)	1.5	TKA	Yes/no	Cemented	LR-PRP; Biomet Biologics GPS	11
Aggarwal AK (2014)	India	RCT	7/14	NR	56.43 (7.59)	53.79 (9.75)	6	TKA	Yes/yes	Cemented	LP-PRP; Immuguard III-PL	8
Morishita M (2014)	Japan	RCT	20/20	2/0	72 (4.1)	74.7 (5.7)	1	TKA	Yes/no	Cemented	LR-PRP; Accelerate Concentrating System	5
Guerreiro JPF (2015)	Brazil	RCT	20/20	6/8	66.4 (9)	71.6 (6.5)	2	TKA	Yes/no	NR	LP-PRP; manual (2 spins)	10
Mochizuki T (2016)	Japan	RCT	109/206	92/106	73 (7.8)	73.4 (8.2)	0.5	TKA	Yes/no	Cemented	NR; manual (1 spin)	5
Evans DC (2018)	United States	RCT	30/30	10/13	NR	NR	1.5	TKA and THA	NR	Cemented	LR-PRP; SmartPrep 2 System	10
Guerreiro JPF (2019)	Brazil	RCT	20/21	7/6	69.14 (6.5)	66.4 (7.25)	24	TKA	NR	NR	LP-PRP; manual (2 spins)	10
Tandogan RN (2020)	Turkey	RCT	40/40	5/4	68 (7)	70 (7)	3 weeks	TKA	Yes/no	Cemented	LR-PRP; Vivostat Processor Unit	4-6
Capion SC (2021)	Denmark	RCT	17/17	3/8	65.6 (8.5)	68.9 (7.1)	4 weeks	THA	NR	NR	LR-PRP; manual (3 spins)	20

*NR, not reported; TKA, total knee arthroplasty; THA, total hip arthroplasty; RCT, randomized controlled trials; PRP, platelet-rich plasma; LP, leukocyte-poor; LR, leukocyte-rich;* *, without the use of commercially available kits.

### Pooled Results

#### Pain

**Figure 3:**
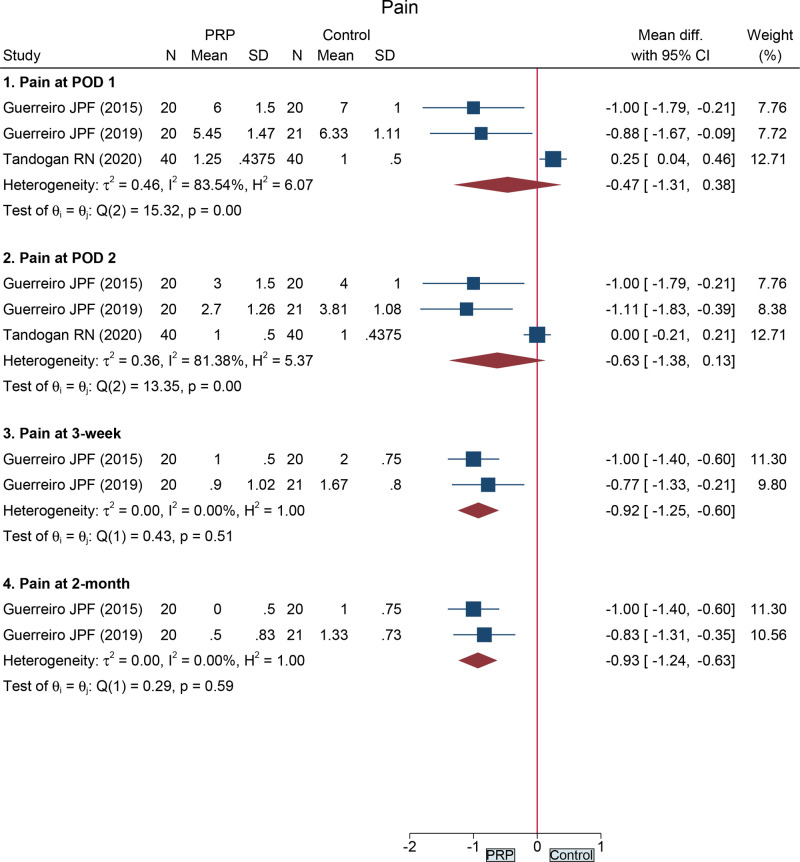
Forest plot presents the pooled results for pain in the total knee arthroplasty subgroup.

A total of 3 studies (16, 18, 30) accessed pain following total knee arthroplasty by using VAS, which included 161 cases. As shown in Figure 3, the PRP group had a similar VAS score to the control group at postoperative day (POD) 1 (MD = −0.47, 95%CI: −1.31 to 0.38), POD 2 (MD = −0.63, 95%CI: −1.38 to 0.13), but 3-week (MD = −0.92, 95%CI: −1.25 to −0.60) and 2-month (MD = −0.93, 95%CI: −1.24 to −0.63) scores were significantly lower than those of the control group.

#### Range of Motion

**Figure 4:**
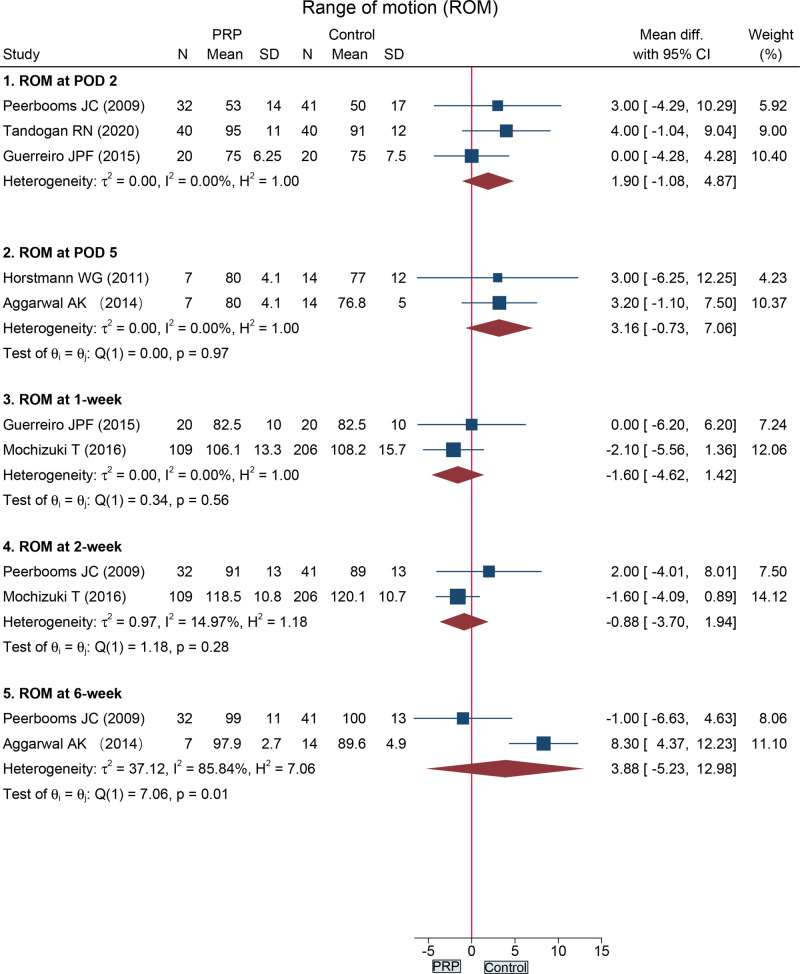
Forest plot presents the pooled results for the range of motion in the total knee arthroplasty subgroup.

Five studies (18, 26, 28, 30, 31) reported ROM following total knee arthroplasty in a total of 529 cases. Between the PRP group and the control group, the ROM was similar at POD 2 (MD = 1.90, 95%CI: −1.08 to 4.87), POD 5 (MD = 3.16, 95%CI: −0.73 to 7.06), 1-week (MD = −1.06, 95%CI: −4.62 to 1.42), 2-week (MD = −0.88, 95%CI: −3.70 to 1.94), and 6-week (MD = 3.88, 95%CI: −5.23 to 12.98) (Figure 4)

#### WOMAC Score

**Figure 5:**
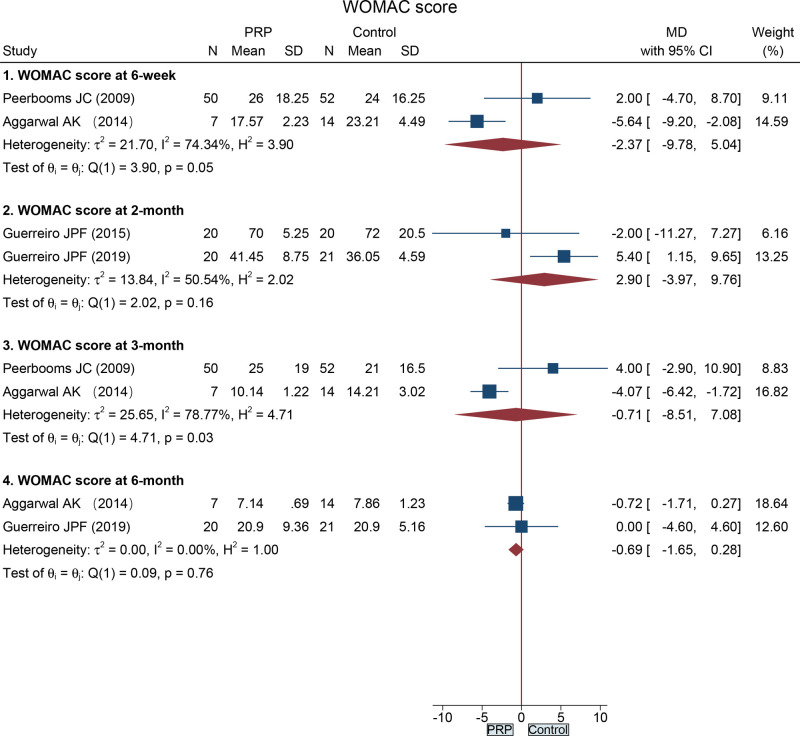
Forest plot presents the pooled results for WOMAC score in the total knee arthroplasty subgroup.

The data on WOMAC score at 6-week, 2-month, 3-month, and 6-months following total knee arthroplasty were recorded in four RCTs (16, 26, 28, 30). The WOMAC score did not significantly differ between the two groups at 6-week (MD = −2.37, 95%CI: −9.78 to 5.04), 2-month (MD = 2.90, 95%CI: −3.97 to 9.76), 3-month (MD = −0.71, 95%CI: −8.51 to 7.08), and 6-month (MD = −0.69, 95%CI: −1.65 to 0.28) (Figure 5).

#### Length of Hospital Stay

**Figure 6:**
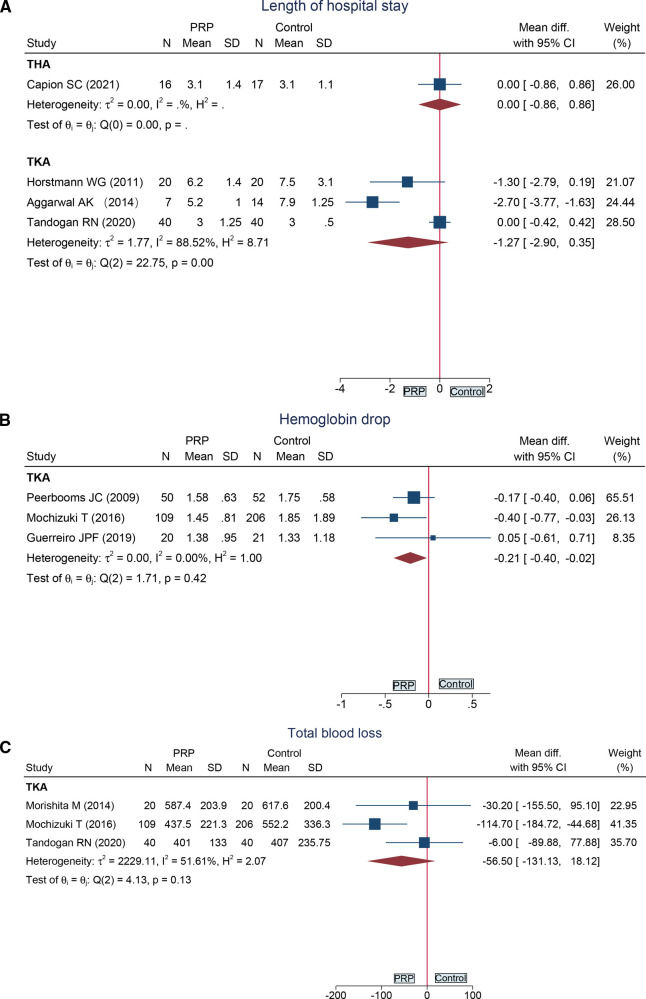
Forest plot presents the pooled results for: (**A**) length of hospital stay; (**B**) hemoglobin drop; (**C**) total blood loss.

Three RCTs (18, 27, 28) reported the length of hospital stay following total knee arthroplasty, and one RCT (33) provided data on total hip arthroplasty. Compared with the control group, no significant difference was found in the THA subgroup (MD = 0.00, 95%CI: −0.86 to 0.86) and TKA subgroup (MD = −1.27, 95%CI: −2.90 to 0.35) (Figure 6A).

#### Hemoglobin Drop at POD 1

Hemoglobin drop following total knee arthroplasty at POD 1 was reported in three RCTs (16, 26, 31) in 458 cases. Compared with the control group, hemoglobin drop was lower in the PRP group (MD = −0.21, 95%CI: −0.40 to −0.02) (Figure 6B).

#### Total Blood Loss

Data from three RCTs (18, 29, 31) demonstrated that total blood loss in the PRP group was similar to that of the control group following total knee arthroplasty (MD = −56.50, 95%CI: −131.13 to 18.12) (Figure 6C).

#### Wound Healing Rate in 4 Weeks

**Figure 7:**
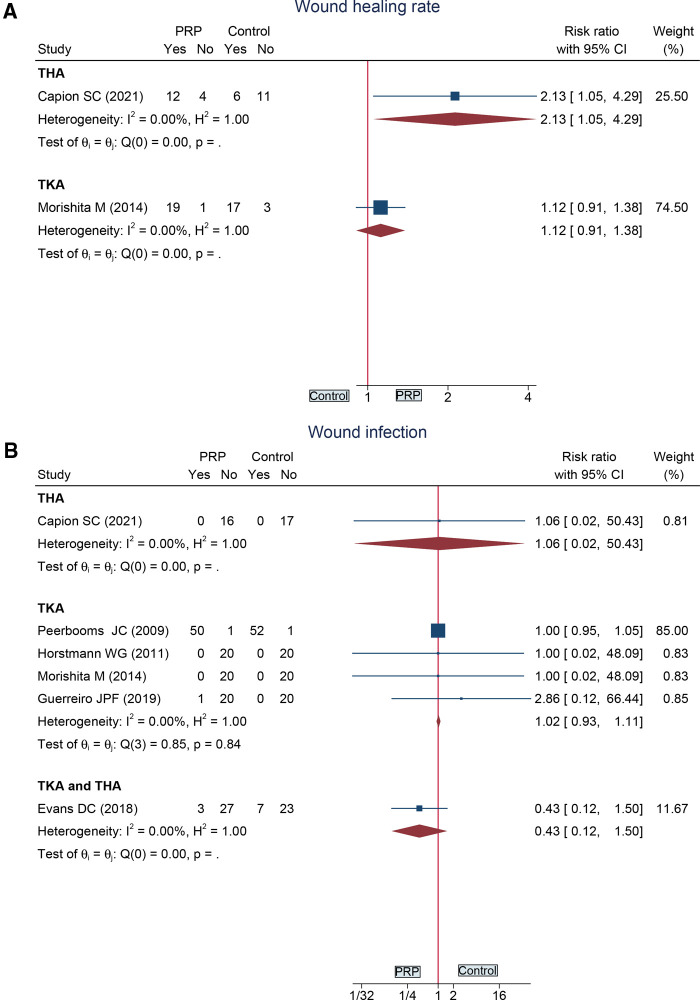
Forest plot presents the pooled results for: (**A**) wound healing rate; (**B**) wound infection.

One study (29) provided total knee arthroplasty data and one (33) provided total hip arthroplasty data on wound healing rates within 4 weeks. There was no significant difference in the TKA subgroup (MD = 1.12, 95%CI: 0.91 to 1.38), while there was a significant difference in the THA subgroup (MD = 2.13, 95%CI: 1.05 to 4.29) (Figure 7A).

#### Wound Infection

Four original studies reported the data of TKA, one study (33) reported the data of THA, and one study (32) reported the data of TKA and THA. However, there was no significant difference in all subgroups (Figure 7B).

#### GRADE Assessment

Table 2 shows the quality of evidence for each outcome. The certainty of pain at 2 months, ROM at POD 5, and WOMAC score at 6 months was high. The level of evidence for ROM at 2 weeks, ROM at 6 weeks, WOMAC score at 6 weeks, and hemoglobin drop was low. The rest of the outcomes were regarded as moderate-level evidence.

**Table 2:** GRADE assessment for outcomes reported in randomized controlled trials (RCTs) on PRP vs control for total knee arthroplasty.

Outcomes	No. of studies	Study design	Risk of bias	Inconsistency	Indirectness	Imprecision	Other considerations	No. of patients		Relative effect (95% CI)	Certainty^a^
								PRP	Control		
Pain at POD 1	3	RCT	No	Serious^c^	No	No	No	80	81	MD −0.47 (−1.31, 0.38)	⊕⊕⊕○ Moderate
Pain at POD 2	3	RCT	No	Serious^c^	No	No	No	80	81	MD −0.63 (−1.38, 0.13)	⊕⊕⊕○ Moderate
Pain at 3 weeks	2	RCT	No	No	No	No	No	40	41	MD −0.92 (−1.25, −0.60)	⊕⊕⊕⊕ High
Pain at 2 months	2	RCT	No	No	No	No	No	40	41	MD −0.93 (−1.24, −0.63)	⊕⊕⊕⊕ High
ROM at POD 2	3	RCT	No	No	No	No	Serious^d^	110	112	MD 1.90 (−1.09, 4.87)	⊕⊕⊕○ Moderate
ROM at POD 5	2	RCT	No	No	No	No	No	27	34	MD 3.16 (−0.73, 7.06)	⊕⊕⊕⊕ High
ROM at 1 week	2	RCT	Serious^b^	No	No	No	No	129	226	MD −1.60 (−4.62, 1.42)	⊕⊕⊕○ Moderate
ROM at 2 weeks	2	RCT	Serious^b^	No	No	No	Serious^d^	159	258	MD −0.88 (−0.37, 1.94)	⊕⊕○○ Low
ROM at 6 weeks	2	RCT	No	Serious^c^	No	No	Serious^d^	70	72	MD 3.88 (−5.23, 12.98)	⊕⊕○○ Low
WOMAC score at 6 weeks	2	RCT	No	Serious^c^	No	No	Serious^d^	57	66	MD −2.37 (−9.78, 5.04)	⊕⊕○○ Low
WOMAC score at 2 months	2	RCT	No	Serious^c^	No	No	No	40	41	MD 2.90 (−3.97, 9.76)	⊕⊕⊕○ Moderate
WOMAC score at 3 months	2	RCT	No	Serious^c^	No	No	No	57	66	MD −0.71 (−8.51, 7.08)	⊕⊕⊕○ Moderate
WOMAC score at 6 months	2	RCT	No	No	No	No	No	27	35	MD −0.69 (−1.65, 0.28)	⊕⊕⊕⊕ High
Length at hospital stay	3	RCT	No	Serious^c^	No	No	No	67	74	MD −1.27 (−2.90, 0.35)	⊕⊕⊕○ Moderate
Hemoglobin drop	3	RCT	Serious^b^	No	No	No	Serious^d^	179	279	MD −0.21 (−0.40, −0.02)	⊕⊕○○ Low
Total blood loss	3	RCT	Serious^b^	No	No	No	No	169	266	MD −56.50 (−131.13, 18.12)	⊕⊕⊕○ Moderate
Wound infection	4	RCT	No	No	No	No	Serious^d^	110	113	RR 1.02 (0.93, 1.11)	⊕⊕⊕○ Moderate

^a^ *GRADE Working Group grades of evidence: High quality = we are very confident that the true effect lies close to that of the estimate of the effect.; Moderate quality = we are moderately confident in the effect estimate: the true effect is likely to be close to the estimate of the effect, but there is a possibility that it is substantially different; Low quality = our confidence in the effect estimate is limited: the true effect may be substantially different from the estimate of the effect; Very low quality = we have very little confidence in the effect estimate: the true effect is likely to be substantially different from the estimate of effect.*

^b^ *Downgraded one level for concerns with performance bias.*

^c^ *Downgraded one level for I^2^ > 50%.*

^d^ *Downgraded one level for publication bias.*

#### Publication Bias and Sensitivity Analysis

Due to the limitations in the number of origin studies, Egger’s test and Begg’s test could not be conducted to assess publication bias. After sensitivity analysis, the results of the current study did not change and were considered stable.

## Discussion

The current systematic review provided the latest evidence involving 772 cases on PRP for TJR. In the TKA subgroup, the available data demonstrated that there was a significant difference in the outcomes of pain and Hb drop, while there was no significant difference in the outcomes for ROM, WOMAC score, LOS, total blood loss, wound healing rate, and wound infection. Moreover, the pooled results found that the PRP group and the control group had similar outcomes of LOS and wound infection in the THA subgroup. Interestingly, data from the included studies revealed that the application of PRP had a positive effect on wound healing rates.

Previous systematic review and meta-analysis including both RCTs and non-RCTs held different points with the current study (15). In this study, Li FX et al included 11 origin studies (7 RCTs, and 4 non-RCTs) to investigate the effect of PRP for TKA, and the pooled results demonstrated that ROM at 3 days and 3 months were significantly higher than in the control group, whereas statistical difference was found in our study. It is noteworthy that non-RCTs included by Li FX et al contributed to bias, which may explain a different point of view from the current study. In another meta-analysis conducted by Ma J et al, which contained 6 RCTs and enrolled 529 patients, it was demonstrated that the application of PRP did decrease the length of hospital stay after TKA (17). However, recently, Capion SC et al (33) found no significant difference in terms of length of hospital stay following TKA between two groups (*P *= .223). Combining the data from Capion SC et al, we found that there was no significant difference in terms of length of hospital stay in the TKA subgroup. For the outcome of hemoglobin drop, a significant difference was found in the study of Ma J et al (17), while Guerreiro JPF et al (16) found no distinction. After pooling the data of Guerreiro JPF et al, the synthesized result of the current meta-analysis remained significant.

Many technologies and drugs have emerged to reduce blood loss during and after total joint replacement (34). It was reported that platelet-rich plasma contributes to reducing blood loss for TJR (17, 28, 31, 35). However, Tingstad EM et al (19) analyzed the data from 93 patients who underwent TKA and discovered that PRP injections did not reduce blood loss (*P *= .686). In a systematic review, Muchedzi TA et al (20) included ten studies to assess average blood loss for patients during TKA, and they also did not find statistical differences between the PRP group and the control group (*P *= .07). Tranexamic acid (TA) administration during TKA had been demonstrated to reduce blood loss (36). Tandogan RN et al (18) evaluated the effect of platelet-rich fibrin (PRF) on blood loss by comparing the combined utilization of PRF and TA with TA alone, and the data showed no significant difference between two groups (*P *= .722). In this meta-analysis, we synthesized the latest evidence and revealed that PRP did not reduce total blood loss in patients following TKA.

Platelet-rich plasma has a strong effect on wound repair (37, 38). A meta-analysis of 15 RCTs by Xia Y et al (14) concluded that additional application of PRP enhanced chronic wound closure. Analogously, the current study provided evidence that PRP accelerates wound healing in total hip arthroplasty. However, there was no significant difference in the total knee arthroplasty subgroup. The reason for the different results may be the differences in cavity between the knee and the hip. A systematic review conducted by Muchedzi TA et al's also found no benefit of PRP application on wound score after TKA (*P *= .33) (20). To the best of our knowledge, this is the first meta-analysis to demonstrate a positive effect of PRP on improving wound rates for patients following THA. Additionally, increased skin healing may be associated with a reduced economic burden. A cost-effectiveness analysis (CEA) conducted by Russo S et al (39) implied that additional PRP was a cost-effective or even a cost-saving alternative treatment for diabetic foot ulcers (incremental cost-effectiveness ratio [ICER] −€613/ QALY). For knee osteoarthritis, however, Rajan PV et al (40) conducted a Markov decision analysis and demonstrated that PRP injections were not cost-effective due to the lack of clinical evidence for pain relief, improved function, and delayed TKA. Here, our study provided the latest data on pain relief for TKA, although the pooled results of postoperative function were not statistically different. Furthermore, the events of wound infection were pooled in the current study, but no difference was found, demonstrating that intraoperative PRP was safe.

Several limitations should be noted. First, only 10 RCTs were extracted in the current meta-analysis. More high-quality RCTs, in the future, are needed to investigate these results. Second, the high heterogeneity should not be ignored. Third, publication bias is a concern, because non-English studies were excluded. Fourth, there is a lack of available data on THA, and only one eligible study was included in this meta-analysis. Therefore, future work should focus on the effect of PRP on THA.

## Conclusion

The application of platelet-rich plasma to patients following total keen arthroplasty is associated with pain relief and decreased Hb drop. In addition, available data reveal that platelet-rich plasma accelerates wound healing rates in patients undergoing total hip arthroplasty. Intra-operative PRP is active; however, more prospective and multicenter studies are warranted to confirm these results.

## Data Availability

The datasets presented in this study can be found in online repositories. The names of the repository/repositories and accession number(s) can be found in the article/Supplementary Material.
